# Post-traumatic stress disorder after first-episode psychosis

**DOI:** 10.1192/j.eurpsy.2021.1212

**Published:** 2021-08-13

**Authors:** M. Pinho, D. Martins, S. Carvalho

**Affiliations:** Department Of Psychiatry, Hospital de Magalhães Lemos, Porto, Portugal

**Keywords:** psychosis, first episode, post-traumatic stress disorder, trauma

## Abstract

**Introduction:**

A psychotic episode may be sufficiently traumatic to induce symptoms of post-traumatic stress disorder (PTSD), which could impact outcomes in first-episode psychosis (FEP). Yet, post-traumatic stress disorder is often left untreated and undiagnosed in the presence of psychosis.

**Objectives:**

To conduct a short review of literature on the prevalence and impact of PTSD after FEP.

**Methods:**

We performed a literature search on PUBMED, using the query: “Stress Disorders, Post-Traumatic” [Mesh] AND “first episode” AND “psychosis”. We focused on data from systematic reviews, clinical trials and meta-analysis published on last 10 years, either in English or Portuguese.

**Results:**

Approximately one in two people experience PTSD symptoms and one in three experience full PTSD, following a FEP. Prevalence may be higher in affective psychosis, inpatient samples and patients previously suffering from depression and anxiety. PTSD Symptom Scale – Self-Report (PSS-SR) can be a useful screening instrument, but there is no established evidence-based intervention for PTSD in people with FEP. Coercive intervention such as involuntary hospitalization, seclusion, restraint or being forced to take medication, as well as being around sick or anxious patients, can be upsetting and traumatizing.

**Conclusions:**

Our data showed high rates of psychosis-related PTSD. To prevent PTSD, conditions of hospitalization should be optimized and the use of coercive treatments should be limited. Subjects with recent-onset psychosis should be screened for PTSD symptoms. Evidence-based interventions to treat PTSD symptoms in the context of FEP are needed to address this burden and improve outcomes.

